# A gelatin-based feed for precise and non-invasive drug delivery to adult zebrafish

**DOI:** 10.1242/jeb.245186

**Published:** 2023-01-30

**Authors:** Aleksander J. Ochocki, Justin W. Kenney

**Affiliations:** Department of Biological Sciences, Wayne State University, Detroit, MI 48202, USA

**Keywords:** Behavior, Drug administration, *Danio rerio*, Pharmacology, Sex differences, Strain

## Abstract

Although the use of adult zebrafish as a model organism has increased in recent years, there is room to refine methods, such as drug delivery, to make them less invasive and more precise. Here, we describe the development of a non-invasive gelatin-based feed method that is tailored to animals based on their body mass. The feed was readily eaten by zebrafish (<1 min) with minimal leaching of compound when placed in water (<5% in 5 min). As a proof of principle, we fed fish a NMDA receptor antagonist (MK-801, 4 mg kg^−1^) prior to the novel tank test. We found that MK-801 caused a general decrease in predator-avoidance/anxiety-like behavior (bottom dwelling) and an increase in locomotion in male fish, but not females. Our simple, easy to prepare and individually tailored gelatin-based feed enables precisely dosed, non-invasive drug delivery to adult-stage zebrafish for the first time.

## Teaser

**Summary:** Development of a new method for precise and non-invasive drug delivery to aquatic animals using a palatable gelatin-based feed.

## Introduction

Zebrafish were first suggested as a model organism in embryology nearly a century ago (Creaser, 1934), although it was not until the 1980s that they became established as a widely used model in developmental biology (Grunwald and Eisen, 2002). Near the turn of the 21st century, zebrafish began to be taken up more broadly in fields such as behavioral pharmacology, neuroscience, immunology and regenerative medicine (Gerlai, 2020; Kenney, 2020; Norton and Bally-Cuif, 2010; Poss et al., 2003; Trede et al., 2004). Given that zebrafish are a relative newcomer to the pantheon of model organisms, there is room to refine methods in areas such as drug delivery (Sneddon et al., 2017), particularly at adult stages.

Pharmacological manipulations in both adult and larval zebrafish have been used to study various aspects of vertebrate biology. Larval fish have the benefit of small size, early life transparency and straightforward drug delivery. Adult animals have the advantage of fully developed organ systems and more sophisticated behaviors (Norton and Bally-Cuif, 2010), but drug delivery is more challenging because of their larger size. Although several methods have been developed for adult animals, each have significant drawbacks. The most common method is beaker dosing, where the drug is dissolved in water and animals are placed in a small beaker (typically 100–250 ml) of solution (e.g. Levin et al., 2006; Montgomery et al., 2010; Sison and Gerlai, 2011). However, the confined space of a beaker is a stressful manipulation (Kalueff et al., 2014) which can interfere with the interpretation of experimental results. Furthermore, the amount of drug uptake is unknown, making it difficult to compare dosing across species. Injection-based methods have also been developed: oral gavage, and intramuscular and intraperitoneal injections (Collymore et al., 2013; Kinkel et al., 2010; Neely et al., 2002). But these methods require anesthesia, which can interact with the drug of interest. They also require more handling and prolonged exposure to the air, which can increase the potential for injury and cause hypoxia, particularly during chronic drug delivery over consecutive days or weeks.

Delivering drugs via feeding has the potential to be both non-invasive and precise. Although a handful of attempts have been made to develop feed-based drug delivery (Chang et al., 2017; Lu and Patton, 2022; Sciarra et al., 2014; Zang et al., 2011), prior approaches have been hampered by the inability to dose animals based on body mass. This is because they rely on either cutting and weighing small amounts of heterogeneous food (Sciarra et al., 2014) or giving the same amount of food to all animals based on an average mass (Chang et al., 2017; Lu and Patton, 2022; Zang et al., 2011), assuming that fish do not vary much in size. To overcome these limitations, we have developed a simple, inexpensive and non-invasive gelatin-based feed method for drug delivery to adult zebrafish that is easily tailored to each animal based on their mass.

## MATERIALS AND METHODS

### Subjects

Subjects were adult male and female AB and TL zebrafish, *Danio rerio* (F. Hamilton 1822), 16–52 weeks of age. All fish used in experiments were bred and raised at Wayne State University. Animals were within two generations of fish originally obtained from the Zebrafish International Resource Center at the University of Oregon. Animals were kept under standard conditions on high-density racks (temperature 27.5±0.5°C, water conductivity 500±10 µS and pH 7.5±0.2) with a 14 h:10 h light:dark cycle (lights on at 08:00 h). Fish were fed twice a day with a dry feed in the morning (Gemma 300, Skretting, Westbrook, ME, USA) and freshly hatched brine shrimp (*Artemia salina*; Brine Shrimp Direct, Ogden, UT, USA) in the afternoon. One week prior to behavioral testing, fish were placed as male/female pairs into 2 l tanks. Tanks were divided in half with a transparent divider, with two fish in each section for a total of four fish in each 2 l tank. Body mass was recorded 1 day prior to experimentation by weighing fish in a beaker containing approximately 50 ml fish facility water. Fish were individually netted and gently patted twice with a dry paper towel to remove excess water prior to weighing. After experiments, animals were euthanized and sex was confirmed by the presence or absence of secondary sex characteristics (i.e. color, shape and fin tubercles) and eggs. All procedures were approved by the Wayne State University Institutional Animal Care and Use Committee.

### Gelatin feed preparation

Our gelatin-based feed was made from a mix of gelatin, spirulina and brine shrimp extract. The brine shrimp extract was prepared by suspending 250 mg ml^−1^ of mikro fine brine shrimp (Brine Shrimp Direct) in water and stirring for 1 h at room temperature. The suspension was centrifuged twice at room temperature at 12,500 ***g*** for 10 min, keeping the supernatant each time. Two volumes of water were then added to dilute the extract, and it was added to a tube containing spirulina (Argent Aquaculture, Redmond, WA, USA) to make a 4% w/v suspension. When drugs were added, part of the diluted extract was replaced with concentrated compound prior to mixing with spirulina to achieve the desired final concentration. To make a 12% w/v gelatin mixture, the suspension was either heated at 45°C for 5 min with periodic vortexing and then added to a tube containing gelatin (Sigma-Aldrich, St Louis, MO, USA) or added to the gelatin at room temperature before heating to 45°C [for dimethylsulfoxide (DMSO; Sigma-Aldrich) and ethanol experiments]. We used a porcine-derived gelatin with a Bloom number of ∼300 g. The mixture was then stored at −20°C overnight. Small morsels for feeding (at 1% body mass) were created by heating gelatin mixture to 45°C until liquid and pipetting onto parafilm. Samples were then placed at −20°C for at least 20 min to re-solidify and kept on ice prior to feeding.

### Methylene Blue leaching

We added Methylene Blue to determine the rate at which compound leaches from our feed. Methylene Blue (Sigma-Aldrich) was added to the feed at a 2 mg ml^−1^ final concentration (equivalent to a 20 mg kg^−1^ dose). Feed samples were made at a volume of 1.75 µl as described above. Samples were placed into 1.5 ml tubes containing 50 µl water and heated to 27°C, the same approximate temperature of our fish facility water, and left for 1, 2.5, 5 or 10 min. At each time point, the supernatant was removed and absorbance at 668 nm (Whang et al., 2009) was read using a NanoDrop 2000C spectrophotometer (version 1.6.198, Thermo Scientific, Waltham, MA, USA). Samples were derived from two separate preparations with three experimental replicates from each set. Absorbance measurements for each experimental replicate were taken in triplicate, and median values were used for analysis. Data were normalized to a sample containing the same concentration of Methylene Blue and brine shrimp extract used in the feed preparation, representing the maximum potential leaching.

### Gelatin-feed administration

To determine whether zebrafish would eat the gelatin feed, we conducted a 5 day trial where our feed was given in lieu of the normal morning feed. Prior to feed administration on each day, fish were transferred from their home rack to a behavioral room and allowed to habituate for 1 h. Transparent barriers were inserted into the tanks, 2–5 min prior to feeding, to briefly isolate fish. Feed was then given to each animal at 1% body mass, an amount we found to yield an appropriately sized morsel that fish could consume easily in one bite. During the trial, we measured the time to eat the feed and whether the feed was successfully eaten within 5 min. Barriers were removed after fish successfully ate the feed, after which they were returned to the housing racks.

### Drug delivery and the novel tank test

As a proof of concept, we used our gelatin-based feed to deliver an *N*-methyl-d-aspartate receptor (NMDAR) antagonist, (+)-MK-801 hydrogen maleate (Sigma-Aldrich), to AB fish prior to capturing their behavior in the novel tank test (Herculano et al., 2015; Menezes et al., 2015; Seibt et al., 2010; Sison and Gerlai, 2011). For each of 2 days prior to drug administration, fish were fed a non-dosed gelatin feed as described above. On the day of behavioral testing, fish were transferred to the behavioral room and allowed to habituate for 1 h. Feed containing MK-801 (4 mg kg^−1^) or vehicle (water) was administered 30 min prior to behavioral testing. The dose was based on prior findings that 2 mg kg^−1^ MK-801 given intraperitoneally altered scototaxis in adult zebrafish (Herculano et al., 2015) and that the oral route results in roughly half the plasma concentration of compound compared with intraperitoneal dosing (Zang et al., 2011). Animals that did not eat the feed were excluded from analysis (2 animals refused the dosed feed and 3 animals were distracted by placement of the barrier and did not eat the gelatin feed during pre-exposure days). For behavior, fish were carefully netted and placed into an open-top frosted acrylic tank (15×15×15 cm, ShopPopDisplays, Woodland Park, NJ, USA) filled with 2.5 l of fish facility water for 6 min. Water was changed between animals. The tanks were kept in a white plasticore enclosure to ensure no disturbances during video recording. Three-dimensional video recordings were captured utilizing D435 Intel Realsense^TM^ cameras (Intel, Santa Clara, CA, USA) mounted 20 cm above the novel tanks, and fish were tracked using DeepLabCut (Mathis et al., 2018) as previously described (Rajput et al., 2022).

### Statistical analysis

Statistical analysis was done using R version 4.1.2 (http://www.R-project.org/), and data were visualized using ggplot2 (Wickham, 2015). ANOVA were performed as described below. Results of behavioral experiments were followed up using false discovery rate (FDR)-corrected *t*-tests within sex (Benjamini and Hochberg, 1995).

## RESULTS AND DISCUSSION

### Development of the gelatin feed

**Fig. 1.**
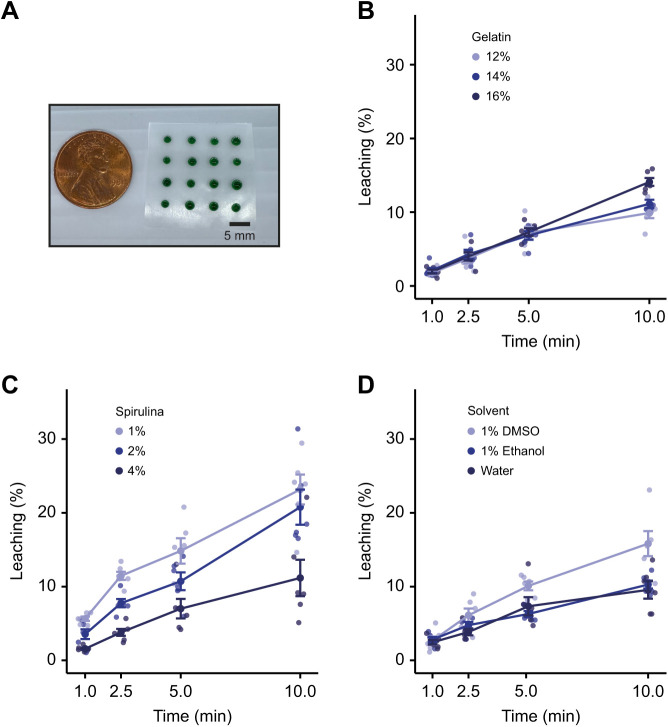
**Preparation of gelatin feed and assessment of leaching.** (A) The gelatin feed (containing Methylene Blue) was pipetted onto Parafilm in individually tailored morsels (1% body mass; shown here against a 1 US cent, 19.05 mm diameter) and allowed to set at −20°C. (B) Methylene Blue leaching over time at different gelatin concentrations with 4% w/v spirulina. (C) Methylene Blue leaching over time at different spirulina concentrations with 12% w/v gelatin. (D) Methylene Blue leaching over time using different solvents (12% w/v gelatin and 4% w/v spirulina). Data are means±s.e.m., *n*=6.

We used gelatin as the base for our feed because of its low melting point and common usage as a food stabilizer. We mixed in spirulina to add color, palatability and nutrition (Khan et al., 2005). When the gelatin-based feed was warmed, we were able to pipette precise volumes of it onto Parafilm before solidification at −20°C (Fig. 1A). Because the feed is administered in an aqueous environment, compound could leach into the water, so we modeled drug loss over time by measuring the release of Methylene Blue dye at different time points (1, 2.5, 5 and 10 min). Initially, we kept the amount of spirulina constant (4% w/v) and varied the gelatin concentration (Fig. 1B). Using a 3×4 (gelatin concentration×time) ANOVA, we found a main effect of time (*F*_3,60_=173, *P*<10^−15^), gelatin (*F*_2,60_=4.6, *P*=0.014) and their interaction (*F*_6,60_=3.63, *P*=0.0039). At 5 min, about 5% of Methylene Blue had leached out for all three concentrations of gelatin, but by 10 min, more Methylene Blue had leached out of the 16% concentration. To determine whether spirulina contributed to leaching, we kept the gelatin concentration constant (12% w/v) and varied the concentration of spirulina (Fig. 1C). A 3×4 (spirulina concentration×time) ANOVA found a main effect of time (*F*_3,60_=60, *P*<10^−15^) and spirulina (*F*_2,60_=32, *P*=2.8×10^−10^) with a trend towards an interaction (*F*_6,60_=1.9, *P*=0.093). Increasing the concentration of spirulina resulted in a clear decrease in leaching that was evident at each time point.

Some drugs may not be soluble in water, instead requiring the use of solvents such as DMSO or ethanol. Because the choice of solvent may affect leaching, we tested the influence of solvent choice in our Methylene Blue assay (Fig. 1D). A 3×4 (solvent×time) ANOVA found a main effect of time (*F*_3,60_=68.8, *P*<10^−15^), solvent (*F*_2,60_=14.7, *P*=6.5×10^−6^) and their interaction (*F*_6,60_=3.23, *P*=0.0081). There was more leaching using DMSO as a solvent, but this was only evident after 5 min. At the 1 and 2.5 min time points, leaching from feed that used DMSO and ethanol as the solvent was similar to that with water (3–5%).

### Gelatin feed palatability

**Fig. 2.**
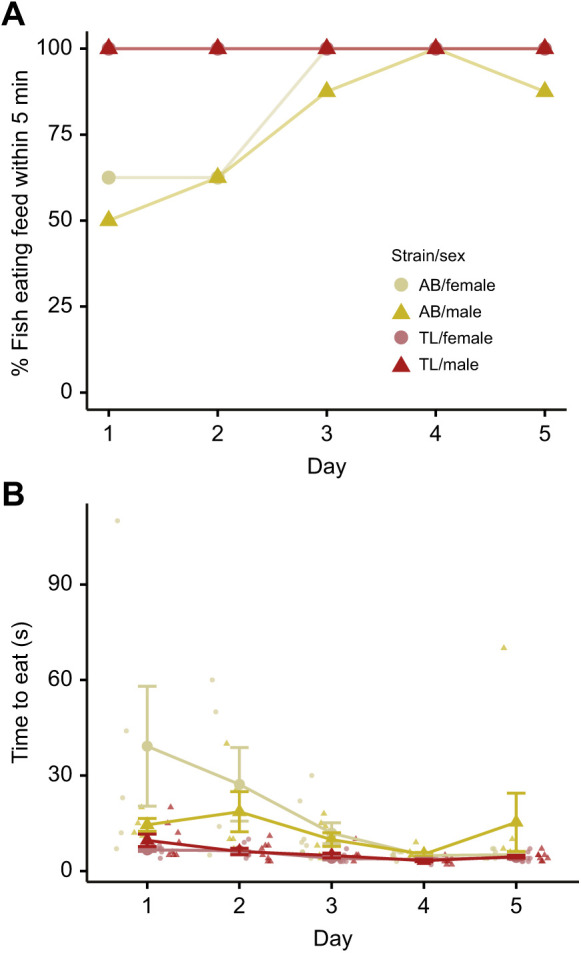
**Eating of gelatin-based feed by fish.** (A) Percentage of AB or TL fish from both sexes that ate the feed within 5 min of administration. (B) Time taken for AB or TL fish of each sex to eat the feed. Fish that did not eat the feed were excluded from time to eat analysis. Data are means±s.e.m., *n*=8 fish per strain/sex.

Next, we sought to determine how readily our feed would be eaten by adult zebrafish. AB or TL fish were given feed (12% w/v gelatin, 4% w/v spirulina) at a volume of 1% body mass for 5 consecutive days in lieu of their normal morning feed (Movie 1). We found that all TL fish ate the feed within 5 min of the very first exposure, but it took 4 consecutive days for 15 of the 16 AB fish to eat consistently (Fig. 2A). For time to eat, a 2×2×5 ANOVA (strain×sex×day) found a main effect of strain (*F*_1,125_=19.4, *P*=2.2×10^−5^) and day (*F*_4,125_=5.80, *P*=0.00026), but no main effect of sex (*F*_1,125_=0.24, *P*=0.63). There were interactions between strain and day (*F*_4,125_=3.35, *P*=0.012), and a strain by day by sex interaction (*F*_4,125_=2.55, *P*=0.04). We found that TL fish, irrespective of sex, ate the food quickly from their first exposure (range: 4–20 s), and this improved to under 10 s for all fish by the fourth day. AB fish initially took longer to eat (day 1 range 7–144 s), with females taking longer than males on the first day, but by the fourth and fifth days, fish of both sexes, except one male AB, ate within 10 s. Thus, after acclimation, fish typically ate the feed quickly (<30 s), which is well before appreciable leaching could occur (Fig. 1).

### Behavioral effect of MK-801 administered using gelatin feed

**Fig. 3.**
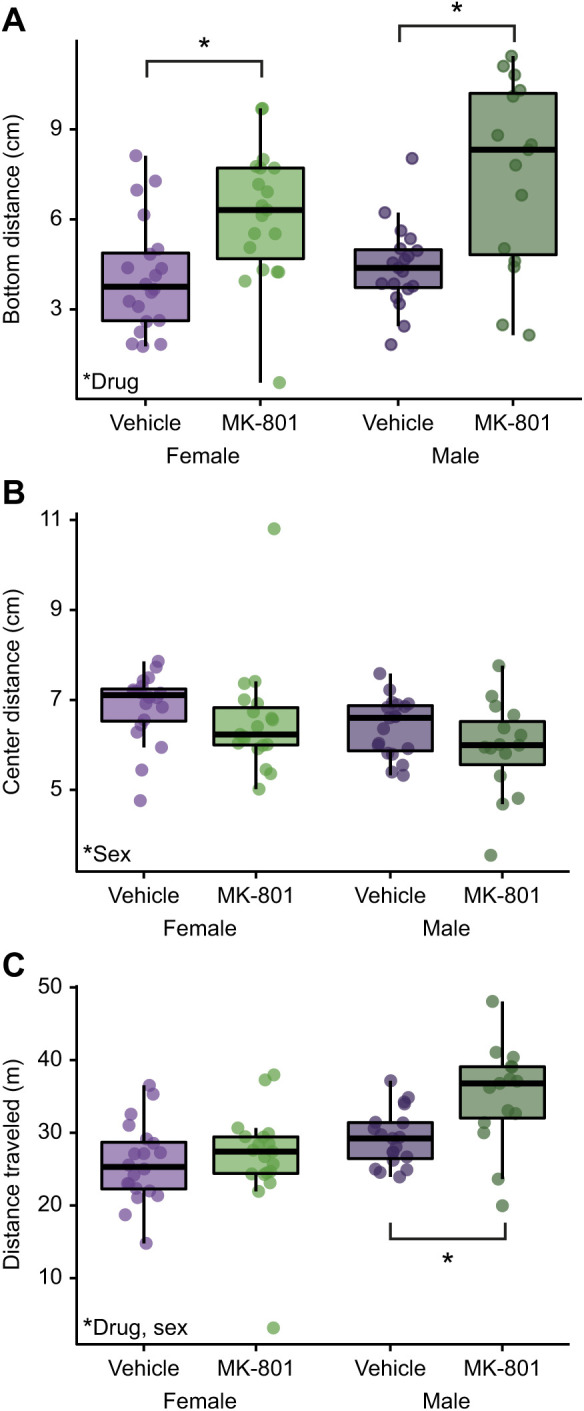
**Behavioral effects of NMDA receptor antagonist administration.** We measured the effect of MK-801 (4 mg kg^−1^) administration 30 min prior to the novel tank test on activity and anxiety-like/predator-avoidance behaviors in AB fish of both sexes. (A) Bottom distance, (B) center distance and (C) distance traveled. Data are presented as box and whisker plots with the median (center line), interquartile range (box ends) and ±1.5 times the interquartile range (whiskers). *P<0.05 based on false discovery rate-corrected *post hoc t*-tests. Female vehicle: *n*=20, female MK-801: *n*=19, male vehicle: *n*=19, male MK-801: *n*=15.

MK-801, an NMDAR antagonist, has been found to alter locomotion and anxiety-like/predator-avoidance behaviors in adult zebrafish (reviewed in Benvenutti et al., 2022). As a proof of principle, we used our gelatin feed to deliver 4 mg kg^−1^ of MK-801 to AB fish 30 min prior to placement in a novel tank. We measured anxiety-like/predator-avoidance behaviors (distance from the bottom and center of the tank) and overall distance traveled (Fig. 3). Statistical significance was assessed using 2×2 (sex×drug) ANOVA. For bottom distance (Fig. 3A), we found a main effect of drug (*F*_1,69_=24.5, *P*=0.000051), where fish given MK-801 spent more time near the top of the tank. There was no effect of sex (*F*_1,69_=2.52, *P*=0.12) or drug by sex interaction (*F*_1,69_=1.0, *P*=0.32). *Post hoc* FDR-corrected *t*-tests within sex confirmed that MK-801 increased bottom distance in both female (*P*=0.0076) and male (*P*=0.023) fish. For center distance, there was an effect of sex (*F*_1,69_=5.84, *P*=0.014), but no interaction between drug and sex (*F*_1,69_=0.76, *P*=0.76), such that males, irrespective of treatment, were closer to the center of the tank than females. There was a trend towards a main effect of drug (*F*_1,69_=3.04, *P*=0.084), where fish given MK-801 appeared to spend more time near the center of the tank. However, FDR-corrected *post hoc t*-tests found no drug effect in either female (*P*=0.35) or male (*P*=0.25) animals. Finally, for distance traveled, there was a main effect of drug (*F*_1,69_=4.2, *P*=0.044), with fish given MK-801 swimming further than vehicle-treated animals. There was also an effect of sex (*F*_1,69_=17.4, *P*=0.000087), with male fish swimming further than females. Finally, there was a trend towards an interaction between drug and sex (*F*_1,69_=3.23, *P*=0.077), such that males appeared to be more affected by the drug than females. FDR-corrected *post hoc t*-tests within sex confirmed the interaction, finding that MK-801 had no effect on distance traveled in females (*P*=0.68) but did in males (*P*=0.012).

### Perspectives

The gelatin-based feed we developed is a simple, precise and non-invasive method for drug administration to adult zebrafish that is an important refinement under the 3Rs ethical framework (Sneddon et al., 2017), minimizing the distress associated with drug delivery. The use of gelatin, which is easily liquified, means that one preparation of feed can be made per drug dose and pipetted into individually sized morsels based on body mass. Finally, as a proof of principle, we used our feed to deliver MK-801, which resulted in increased locomotor activity and decreased predator-avoidance/anxiety-like behaviors, consistent with prior work (Benvenutti et al., 2022).

Our gelatin feed is an important improvement in terms of precision and ease of use compared with other feed-based drug delivery strategies that have been developed for adult zebrafish. For example, Sciarra and colleagues (2014) described the use of a commercial gelatin-based feed, Gelly Belly, for drug delivery. However, precise drug delivery with Gelly Belly is difficult because the food is inhomogeneous and requires cutting and weighing small amounts of solidified food for each animal. Other approaches are not easily tailored to individual fish based on body mass, such as a gluten-based feed described by Zang and colleagues (2011) or a gelatin/agar paste that is pressed into a 3D printed mold (Lu and Patton, 2022). These approaches administer the same amount to each fish, relying on the assumption that all animals are the same mass. However, we and others have found that fish vary considerably in size even when they are the same age. For example, in the present study, the average mass of our fish was ∼252±50 mg (mean±s.d.) with a range of 155 to 435 mg. This means a drug dose developed for the average body mass would result in a 60% overdose of our smallest fish and a 40% underdose of our largest fish. Notably, the minimum and maximum body masses (i.e. 155 and 435 mg) were observed in fish of the same age, sex and strain. The variability we observed is similar to other reports: our standard deviation (i.e. ∼20% of average mass) is lower than, or similar to, other studies that use fish of the same age (e.g. Fowler et al., 2019; Siccardi et al., 2009).

One interesting finding in the present work was that MK-801 induced an increase in locomotor activity in male, but not female, fish. To our knowledge, this is the first report of a sexual dimorphism in response to NMDAR antagonism in zebrafish, although sex differences in response to MK-801 have been found in rodents (Hönack and Löscher, 1993; Lipa and Kavaliers, 1990). The selective effect of MK-801 on male fish is likely due to sexual dimorphism in the functioning of the glutamatergic system (Wickens et al., 2018), but the exact mechanism remains to be uncovered.

Some drawbacks to feed-based methods for drug delivery are that they require the drug to cross the intestines, the drug can leach from the feed or the animals may refuse the feed if the taste of a drug is unpalatable. Palatability can be overcome by using attractants or other additions to the feed to mask the taste. For example, additions such as clam juice (Chang et al., 2017; Sciarra et al., 2014) or Power Bait, a commercial fish attractant (Lepage et al., 2005), have been successfully used to overcome the taste of added compounds. Here, we used an extract of freeze-dried brine shrimp. Another option would be to lower the drug dose and feed fish multiple boluses to reach the appropriate dose. With respect to drug leaching, within the time frame in which fish consume the feed, we found negligible loss of compound. One limitation of our experiments is that we only tested leaching with Methylene Blue; other compounds with different chemical structures may leach more or less slowly.

### Conclusion

Overall, our gelatin-based feed method is an important refinement for the administration of drugs to adult zebrafish. It increases precision and avoids the stress associated with techniques such as beaker dosing or injection. Because our feed can be easily pipetted into individually tailored morsels, drug delivery is based on body mass, enabling direct comparison of drug doses across species. Taken together, our new method improves the utility of adult zebrafish for understanding vertebrate biology.

## Supplementary Material

10.1242/jexbio.245186_sup1 Supplementary information
