# Supplementary figures and images for: A gelatin-based feed for precise and non-invasive drug delivery to adult zebrafish
Source: J Exp Biol. 2023 Jan 30;226(2):jeb245186. doi: 10.1242/jeb.245186 (PMC10165467; doi:10.1242/jeb.245186)

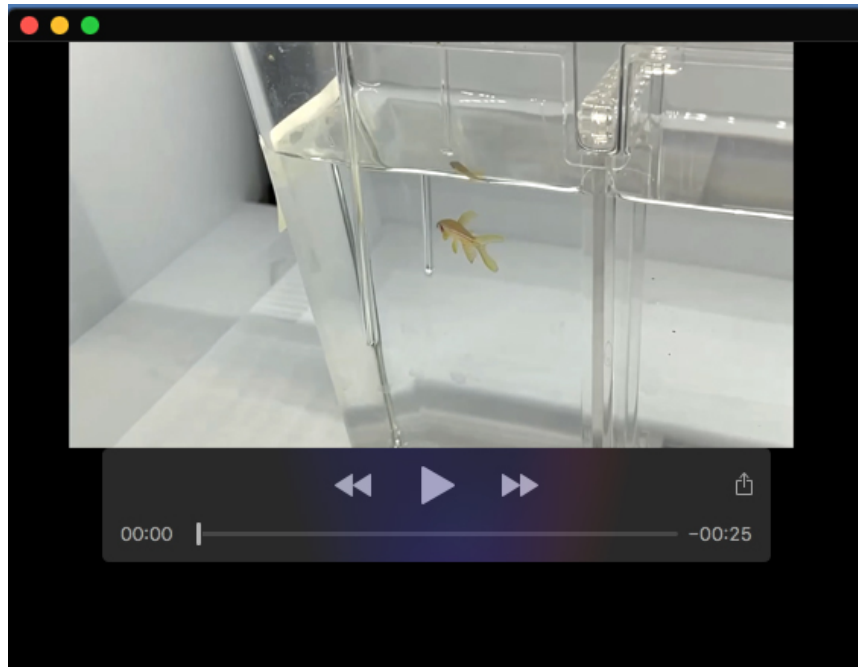

**Movie 1.** Video of TL zebrafish rapidly consuming the gelatin-based feed.

Supplement: Supplementary information [file jexbio-226-245186-s1.pdf]
